# cGAS-STING pathway in oncogenesis and cancer therapeutics

**DOI:** 10.18632/oncotarget.27673

**Published:** 2020-07-28

**Authors:** Brandon Yi Da Hoong, Yunn Hwen Gan, Haiyan Liu, Ee Sin Chen

**Affiliations:** ^1^Department of Biochemistry, Yong Loo Lin School of Medicine, National University of Singapore, Singapore; ^2^National University Health System (NUHS), Singapore; ^3^Wong Hock Boon Society, Yong Loo Lin School of Medicine, National University of Singapore, Singapore; ^4^Department of Microbiology and Immunology, Yong Loo Lin School of Medicine, National University of Singapore, Singapore; ^5^NUS Graduate School of Integrative Sciences & Engineering (NGS), National University of Singapore, Singapore

**Keywords:** cyclic GMP-AMP synthase, stimulator of interferon, cGAS, STING, interferon

## Abstract

The host innate immunity offers the first line of defense against infection. However, recent evidence shows that the host innate immunity is also critical in sensing the presence of cytoplasmic DNA derived from genomic instability events, such as DNA damage and defective cell cycle progression. This is achieved through the cyclic GMP-AMP synthase (cGAS)/Stimulator of interferon (IFN) genes (STING) pathway. Here we discuss recent insights into the regulation of this pathway in cancer immunosurveillance, and the downstream signaling cascades that coordinate immune cell recruitment to the tumor microenvironment to destroy transformed cells through cellular senescence or cell death programs. Its central role in immunosurveillance positions the cGAS-STING pathway as an attractive anti-cancer immunotherapeutic drug target for chemical agonists or vaccine adjuvants and suggests a key node to be targeted in a synthetic lethal approach. We also discuss adaptive mechanisms used by cancer cells to circumvent cGAS-STING signaling and present evidence linking chronic cGAS-STING activation to inflammation-induced carcinogenesis, cautioning against the use of activating the cGAS-STING pathway as an anti-tumor immunotherapy. A deeper mechanistic understanding of the cGAS-STING pathway will aid in the identification of potentially efficacious anti-cancer therapeutic targets.

## INTRODUCTION

When functioning properly, the immune system protects the body against disease and pathogenic attack. The first line of defense is the innate immune system, which provides nonspecific defense mechanisms, including physical, chemical and immune responses, which protect the organism against microbial insult. Specialized RNA- and DNA-sensing receptors police subcellular compartments for infiltrating foreign nucleic acids, and, upon recognition, trigger immune signaling pathways that initiate a host defense. DNA infiltration into the cytosol can occur following a range of cellular changes or insults, including cellular senescence [1, 2], replication stress [3], mitochondrial stress [4], or other cytosolic pathogens or insults, such as irradiation [5]. These changes lead to the production of type I interferon (IFN) [6], which helps to activate the second line of defense, the adaptive immune response. Elicited by lymphocytes, the adaptive immunity coordinates a more long-term response that helps to restore homeostasis.

**Figure 1:**
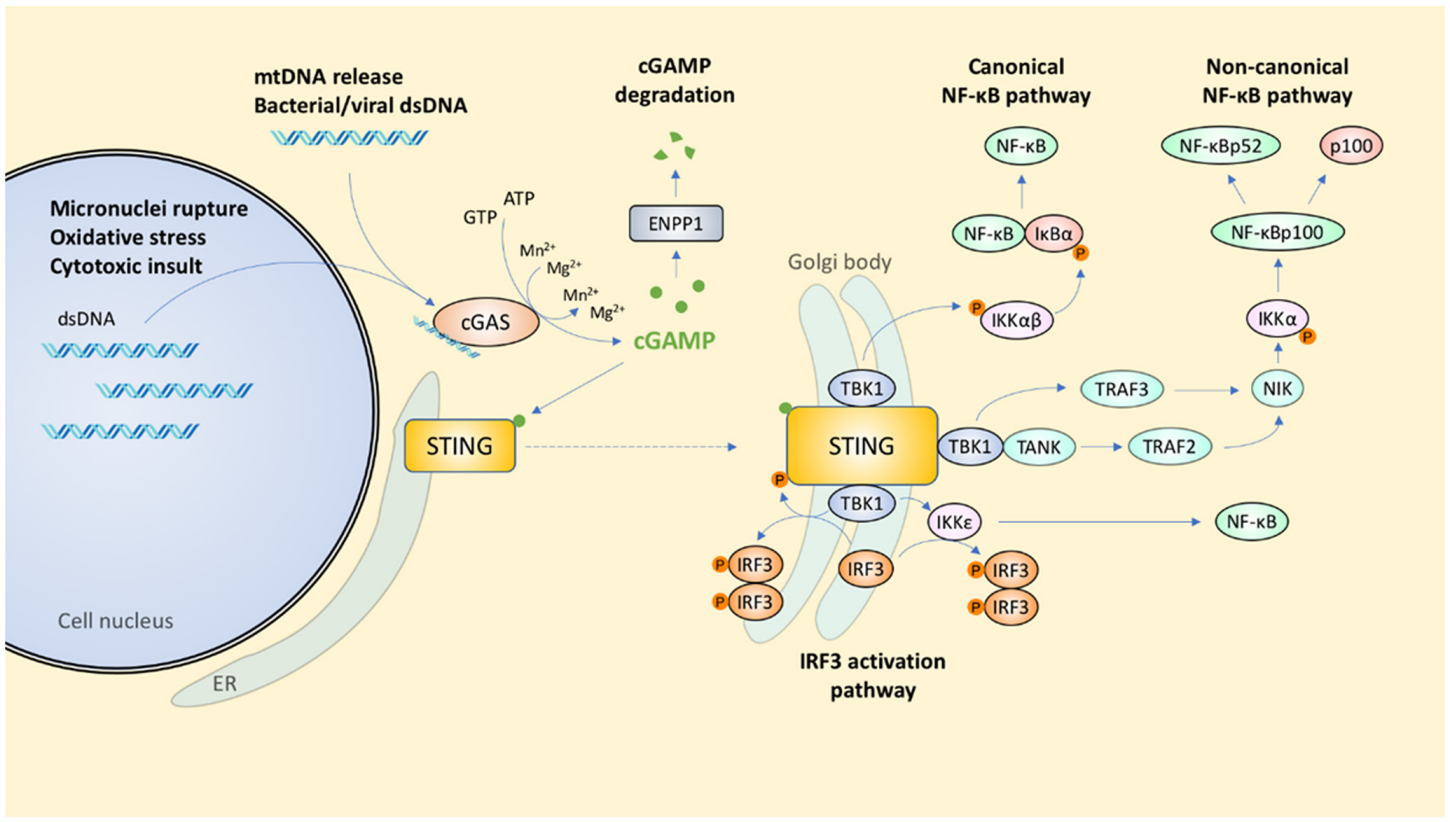
Signal transduction coordinated by the cGAS-STING pathway. Innate immunity is triggered by the escape of nuclear or mitochondrial dsDNA into the cytosol.

The innate immune system is triggered by the presence of nucleic acids in the cytoplasm through a range of pattern recognition receptors (PRRs) [7], including Toll-like receptors (TLRs), nucleotide-binding oligomerization domain (NOD)-like receptors, cytosolic DNA sensors, retinoid acid-inducible gene I (RIG-I)-like helicases [7–11], absent in melanoma 2 (AIM2), DNA-dependent activator of IFN regulatory factors (DAI), DEAD (Asp-Glu-Ala-Asp) box polypeptide 41 (DDX41), IFNγ-inducible protein 16 (IFI16), Sm-like (LSm) 14A (LSm14A) and Sry-related high-mobility group (HMG) box 2 (Sox2) [12–17] and cyclic GMP-AMP synthase (cGAS)/stimulator of interferon (IFN) genes (STING) pathway [6] (Figure 1). These various PRRs present on host immune cells sample the cellular milieu for danger- and pathogen-associated molecular patterns present on pathogens. Of the PRRs described, this review will focus on the cGAS/STING pathway, a pathway first identified as a signaling pathway activated by double-stranded DNA (dsDNA) during pathogenic infection. More recently, studies have suggested that the cGAS-STING pathway may be an ideal target for therapeutic intervention. To explore this further, here we describe the cGAS-STING signaling cascade and its extensive downstream activation channels, highlight some of the triggers for pathway activation, outline its diverse roles in carcinogenesis and anti-tumor immunity, and proposes some implications for its potential role in anti-cancer therapy.

## ENDOGENOUS DNA ACTIVATES THE cGAS-STING PATHWAY

### cGAS produces cGAMP upon association with dsDNA

cGAS is a mediator of the DNA sensing pathway that is activated upon the presence of DNA in the cellular cytosol. cGAS is an enzyme of the nucleotidyltransferase family and senses the aberrant expression of viral and bacterial dsDNA within the cytosol in a sequence-independent manner. Binding of cGAS to dsDNA promotes cGAS oligomerization and activation, which catalyzes the formation of the cyclic dinucleotide (CDN), cyclic GMP-AMP (cyclic [G (2’,5′) pA (3′,5′) p]; 2’3′-cGAMP), otherwise referred to as cGAMP [18–21]. cGAS contains two dsDNA binding sites that bind DNA cooperatively [22]. The interaction between cGAS and DNA induces cGAS dimerization and triggers a conformational change that unveils an entry point for ATP and GTP to bind to an internal catalytic pocket [19, 22]. The subsequent formation of phosphodiester bonds between ATP and GTP then produces cGAMP [19].

Not all types of nucleic acids are ligands for cGAS–RNA:DNA hybrids and the cytosolic B-form of dsDNA will trigger cGAS activity [23, 24], whereas dsRNA, ssRNA, and ssDNA do not induce any cGAMP formation; albeit, dsRNA can bind cGAS [24]. The dsDNA ligands may be nuclear or mitochondrial, but not endosomal [18], and the interaction between cGAS and dsDNA is predominantly sequence-independent, involving hydrogen bonding on the sugar-phosphate backbone of DNA [19] as well as electrostatic interactions [22]. cGAS activity also depends on several other factors, including the concentrations of enzymes, substrates, ionic cofactors, and environmental salt. Likewise, the lengths of the dsDNA substrates, and any post-translational modifications on cGAS will affect the degree of cGAS activation [25–27]. For instance, a phase transition occurs when the physiological ion concentration of the negatively charged DNA and the positively charged N-terminus of cGAS both exceed 30 nM [25]. This leads to the formation of liquid droplets, which enhance the local substrate-enzyme concentration and facilitate the catalysis of cGAMP [25]. Although the binding affinity of cGAS-DNA is enhanced in low-salt environments, the presence of specific ions, especially Zn^2+^ and Mn^2+^, can stimulate cGAS activity [25]. However, the generalizability of these findings to cells *in vivo* is uncertain as the effect of ionic concentrations were demonstrated either in buffered cell-free *in vitro* environments or with the aid of specific ion chelators. Additionally, there is limited literature documenting the variation in ionic concentrations in different cell types *in vivo*, including cancer cells, possibly due to the tight ionic regulation of the intra- and extracellular compartments for homeostasis. The valences of cGAS and dsDNA also determine the strength of binding and, consequently, the enzymatic activity of cGAS; indeed, larger dsDNA moieties induce a greater accumulation of cGAMP with increased potency [25, 27]. Generally, however, in human cells, dsDNA larger than 70-bp is required for efficient cGAS-STING activation [28].

Finally, cGAS activity is also affected by transcriptional regulation and post-translational regulatory controls. The transcriptional factors Sp1 and CREB can bind to the promoter region of the cGAS gene to facilitate transcription [29]. The acetylation of cGAS at residues K384, K394, and K414 can render cGAS inactive, whereas stimulation with exogenous DNA causes cGAS deacetylation by the histone deacetylase HDAC3 prior to cGAS pathway activation [26]. cGAMP is degraded by the type II transmembrane glycoprotein, ecto-nucleotide pyrophosphate phosphodiesterase 1 (ENPP1), which normally hydrolyses ATP to from AMP and diphosphate [30] (Figure 1).

### Second messengers and STING activation

Cyclic dinucleotides (CDNs)—such as c-di-GMP and cGAMP—serve as second messengers for endoplasmic reticulum (ER)-bound STING [31, 32]. 2’3′-cGAMP is a higher affinity ligand for STING as compared with other CDNs (3′3′, 2′2′, 3′2′-cGAMP, or c-di-GMP) by virtue of the extensive polar and hydrophobic interactions between 2′3′-cGAMP and the STING binding site [20]. Whereas human STING can still recognize bacterially derived CDN, certain naturally occurring polymorphisms of STING, such as R232H, confer structural selectivity to non-canonical CDNs (e.g., 2′-3′ cGAMP) [21]. It is interesting to also note that STING can be directly stimulated by many CDNs that are second messengers produced by bacteria for varied processes including regulation of virulence, biofilm formation and sporulation [31, 33, 34]. Furthermore, cGAS-STING has also been reported to be activated by CDNs in an DNA-independent manner [35, 36].

Binding of CDNs to STING leads to its translocation from the ER to the Golgi [37], where it is activated by palmitoylation on Cys88 and Cys91 [38–40]. Previously, it was thought that STING complexes with TANK-binding kinase 1 (TBK1; also called NF-κB-activating kinase [NAK]) before trafficking from the ER to Sec5-containing endosomal compartments *via* the Golgi [37]; however, it has since been shown that translocation to the Golgi is a prerequisite for STING to bind TBK1 [41]. TBK1 is recruited to a conserved PLPLRT/SD motif in the C-terminal tail of STING [42], with IFIT3 serving as an adaptor bridging TBK1 to STING [43]. Having overcome geometric constraints found in TBK1, further higher order oligomerization of the STING-TBK1 complex facilitates TBK1 trans-autophosphorylation and STING phosphorylation by TBK1 [42, 44]. Both Cys91 palmitoylation and the α2-α3 loop of the ligand binding domain are implicated in STING oligomerization [39, 45]. Consistent with this, small molecular inhibition of Cys91 palmitoylation using the C-178 nitrofuran inhibits cGAMP-induced STING oligomerization and translocation, and downstream IFNβ production [39]. Mutations in the α2-α3 loop interfacing residues Q273A and A277Q also yields the same effect [45]. At the Golgi, the STING-TBK1 interaction eventually activates interferon regulatory factor 3 (IRF3) and NF-κB [46, 47], two major effectors of innate immunity (Figure 1).

### Micronuclei formation and breakdown

At the end of mitosis, mis-segregated chromosomes can become separated from the main chromatin mass and encased in their own nuclear envelopes, forming “micronuclei” [48], which are regarded as hallmarks of genomic instability. Irradiation and other external insults can similarly cause the formation of micronuclei [5]. Similarly, genomic instability brought about by, for example, the selective deletion of the ribonucleotide excision repair enzyme RNase H2 in mouse embryonic fibroblasts (MEFs) can also trigger micronuclei formation [5, 49].

Micronuclei are highly susceptible to irreversible membrane rupture due to a structural defect in the organization of the lamina; specifically, a lower expression of lamin B1 as compared with the normal nuclear envelope [48]. However, it is unclear why such distinctions in the lamina structure even occur. What does appear to be consistent is that nuclear envelope collapse is a typical consequence of micronuclei formation, and it happens in tumors, immortalized epithelial cells, and primary fibroblasts [48]. Lamin B1 expression is also found to be lower in senescent cells, causing chromatin fragment release and the potent activation of cGAS [50, 51]. MacKenzie *et al*. proposed that cGAS localizes to micronuclei following rupture of the micronuclear envelope [5], and the subsequent release of these fragments into the cytosol induces a cGAS-mediated inflammatory response.

The formation of micronuclei appears to be cell cycle dependent as it is greatly augmented by passage through the mitotic phase of the cell cycle, which results in the formation of chromatin bridges and mis-segregated chromosomes [1, 5, 52, 53]. cGAS knockdown in combination with mitotic arrest increased micronuclei formation and chromosomal segregation defects, whereas inhibition of the mitotic inducer cyclin dependent kinase 1 (CDK1) or overexpression of the cell cycle inhibitor p21, which inhibits the transition of cells from G2 into the mitotic phase, reduced micronuclei formation [53, 54]. Furthermore, despite hosting damaged DNA, cells that exit mitotic cell cycle into G0 stage showed impaired micronuclei formation, lending further support to micronuclei as the products of mitosis [50] cGAS can be localized to the foci of nuclear envelope disruption [49], and the rupture of the micronuclei spills genomic DNA into the cytosol, resulting in cGAS-STING activation [55, 56]. Interestingly, cGAS can also accumulate at micronuclei bodies to activate downstream proinflammatory responses within the micronuclei [57, 58], even though the mechanisms underlying this translocation process remains poorly defined [5, 58, 59]. These results indicate that cell cycle progression is required to activate innate immune sensing *via* the cGAS-STING pathway when DNA is damaged.

### DNA replication stress and mitotic changes

Replication stress can also contribute to an increase in cytosolic DNA, and can arise from exposure to genotoxic agents (for examples, arabinofuranoside [3] and etoposide [60]), or simply following the replication of intrinsically difficult DNA sequences [61]. Cytosolic DNA contains retroelements and is derived from the parts of genomic DNA that are predisposed to forming non-B form DNA structures, including R-loops [3]. These structures inhibit the progression of DNA replication, causing the replication fork to stall and collapse, which can simultaneously lead to the production of DSBs and ssDNA extension [62]. Stalling of the replication fork can also lead to an accumulation of genomic DNA in the cytoplasm, with subsequent cGAS activation and type I IFN production [3, 60]. RNase H2, a ribonucleotide excision repair enzyme [63], degrades RNA:DNA found in R-loops and active reverse-transcribed retroelements [64]. Recent work found that overexpression of RNase H1 led to a reduction in cytosolic DNA accumulation [3], and RNase H2-deficient mice had increased micronuclei formation and cGAS stimulation [5, 65]. It is therefore possible that RNA:DNA hybrid accumulation can also trigger cGAS activation; albeit, how this happens remains unclear [65]. Collectively, these findings point to the important role of replication stress in the release of nuclear DNA into the cytoplasm.

cGAS localizes to nuclear chromatin during mitosis and dissociates at the end of mitosis [1, 58, 66]. However, cGAS is prevented from activating pro-inflammatory signaling during mitosis not only because of the transient nature of mitosis (30 minutes) compared with cGAS signaling (hours), but because of the dense compaction of DNA into chromosomes and the attachment of peri-chromosomal proteins [5]. In addition, cGAS-STING pathway genes could also be transcriptionally silenced during mitosis despite the ubiquity of DNA in the cytoplasm, thereby preventing expression of interferon-stimulated genes (ISGs) [5]. A recent study found that while cGAS localizes to chromosomes immediately after nuclear envelope breakdown, their higher binding affinity for nucleosomes compared to naked DNA can suppress DNA-induced cGAS signaling [66]. Regardless, because STING remains in the cytoplasm throughout mitosis [1], the colocalization of cGAS and nuclear chromatin suggests that cGAS could play a role in cell cycle regulation in a STING-independent manner. Further studies are required to fully understand the role of cGAS in mitosis.

### Nuclease activities

Nucleases encompass a broad class of enzymes that play critical roles in DNA proofreading and repairs during replication, and nucleotide turnover. Nucleases hydrolyze the phosphodiester bonds of DNA and RNA yet show selectivity for certain types of nucleic acids: endonucleases variably cleave DNA and RNA within the strand, whereas exonucleases selectively degrade single-stranded DNA and remove 3′ or 5′ overhangs.

Intriguingly it appears that both DNA damage and the ensuing response to DNA damage can trigger cGAS-STING-mediated type I IFN production. One of the DNA damage responses is mediated by the MUS81 endonuclease, which cleaves non-B DNA structures at stalled replication forks [67]. This reduces chromosomal instability but leads to the generation of cytosolic DNA, the secretion of type I IFN, and ultimately cGAS/STING activity [68]. However, in the event of replication fork stalling, SAMHD1, a dNTPase, activates the MRE11 exonuclease to prevent the release of genomic DNA into the cytoplasm, thereby inhibiting the proinflammatory response and, by extension, cGAS/STING activation [55]. Yet, others show that nuclear DNA damage induces the nuclear translocation of cGAS, where it associates with PARP1 at DSBs [69]. As a result, PARP1-Timeless complex formation is impeded and this prevents homologous recombination-dependent DNA repair in an IFNβ-independent manner [69].

Trex1 (DNaseIII) is another nuclease that has been linked with cGAS activation. Trex1 is a widely distributed exonuclease that degrades cytoplasmic DNA. Therefore, it is not surprising that the absence of Trex1 can increase cytosolic DNA levels and augment the cGAS-STING inflammatory response [60, 70]. Interestingly, Trex1 was also found to translocate to the nucleus following DNA damage where it interacts with and stabilizes PARP1 to facilitate the DNA damage response [71]. Trex1 thus likely acts to prevent any uncontrolled inflammatory responses in response to DNA damage or leaked DNA in a healthy cell. Oxidized cytosolic DNA, however, is more resistant to degradation by Trex1, leading to cytoplasmic DNA accumulation and a cGAS-STING-mediated inflammatory response [72]. Similarly, impaired RNase H2 expression, which normally suppresses the DNA damage immune response by degrading RNA:DNA hybrids, leads to an increase in cytosolic DNA, while the overexpression of RNase H1 reduced type I IFN production [3, 5].

### Other types of DNA

Damage to mitochondrial DNA (mtDNA) also contributes to cGAS activation in the context of cellular stress. Unlike nuclear DNA, mtDNA is not protected by histones and is thus more susceptible to damage [73]. Mitochondrial stress also generates ROS [74], resulting in mtDNA oxidation and degradation [75]. For example, under hypoxic conditions, breast cancer cells experience mitochondrial fragmentation and mitophagy. Such damaged mitochondria are targeted for degradation, releasing mtDNA into the cytoplasm, and inducing cGAS activation and downstream IFN-β production [4]. Neutrophil extracellular traps (NETs) containing oxidized neutrophilic mtDNA can also trigger circulating peripheral blood mononuclear cells (PBMCs) to produce IFN *via* the cGAS-STING pathway [76]. Cytosolic mtDNA acts as a ligand for further cGAS-STING activation that potentiates caspase-independent, immunogenic (IFN-mediated) cell death [77].

### Intercellular transfer of cGAMP

cGAMP can stimulate STING translocation and IRF3 phosphorylation in adjacent cells through gap junctions [78]. This transfer effect is abrogated upon separation of cells or knockdown of connexin, a component of gap junctions [78]. It is highly plausible that loss of cell–cell adhesion in malignant cell clusters can compromise this mechanism of immune-mediated clearance. Indeed, brain metastases from lung and breast cancer depend on the formation of gap junctions with brain astrocytes *via* Cx43 connexin molecules with the assistance of PCDH7 following extravasation from blood vessels [79]. These cancer cells transfer cGAMP—produced by cGAS in response to tumor cytosolic DNA—to astrocytes by establishing gap junctions, thereby activating STING and inducing type I IFN and TNFα expression [79]. Secreted IFN and TNF act as paracrine signals to trigger STAT1 and NF-κB signaling in metastatic cells to promote cell survival and facilitating and maintaining brain metastasis [79]. Intriguingly, this pro-metastatic response to STING activation is in contrast with the anti-tumor effects observed with short-term STING-mediated innate immunity, yet both have a similar cytokine profile. While the specific tissue context within which STING is activated could be a contributory factor, more work is needed to elucidate the role of the tissue environmental niche on the STING immune response.

Viral particles also mediate a form of intercellular cGAMP transfer. Viral particles package cGAMP from infected host cells and transfer it to newly infected target cells. This triggers a STING-dependent type I IFN reporter release by the host as a defense mechanism against the infectious pathogen [80, 81]. The viral transfer of cGAMP to dendritic cells (DCs) also initiates an innate immune response [81], offering an alternative exploitable mechanism for the anti-tumor activity of oncolytic viruses.

More recently, the SLC19A1 protein, also known as reduced folate carrier 1 (RFC1), was identified as the first known transmembrane transporter of extracellular cGAMP and other CDNs [82, 83]. The authors showed that SLC19A1 contributed to the uptake of cGAMP by hematologic cell lines, but found that this activity was variable among PBMCs derived from different donors [82, 83]. Understanding the alternative mechanisms of intercellular cGAMP transfer, uptake and degradation will be important for the development of novel therapeutics in this area. The identification of SLC19A1 as the major transporter of CDN uptake in humans, and its inhibition by folates and certain anti-inflammatory drugs, suggests that SLC19A1 expression could play a pivotal role in predicting responsiveness of tumors to CDN stimulation [83]. Indeed, studies have shown that the chemotherapy drug carboplatin combined with gap junction modulators meclofenamate and tonabersat can block intercellular cGAMP transfer resulting in better control of brain metastasis *in vivo* [79].

## DOWNSTREAM EFFECTORS OF cGAS-STING SIGNALING

### cGAS responds to cellular damage by inducing type I IFN production

Type I IFNs secreted by infected cells have three major functions: they limit the spread of infection by inducing innate antimicrobial activity in infected and neighboring cells; they promote antigen presentation and NK cell functions while controlling pro-inflammatory pathways; and they activate adaptive immunity *via* T and B cell responses.

cGAS is essential for IFN-β expression, as demonstrated by the inability of cGAS-knockout mice to release type I IFN after detecting cytosolic DNA [84]. There are several ways in which STING can regulate downstream transcription factors for type I IFN production. STING can activate TBK1 *via* the E3 ubiquitin ligase TNF-receptor associated factor 6 (TRAF6)-dependent pathway to activate NF-κB, which is then translocated to the nucleus to activate IFNβ expression [85]. STING can also activate NF-κB *via* IKKε and IRF3 [86]. The physical interaction of STING with both TBK1 and IRF3 facilitates the phosphorylation of STING and IRF3 by TBK1 [42, 47, 87], which in turn results in IRF3 dimerization and its subsequent nuclear translocation [88] (Figure 1).

NF-κB and IRF3 transactivate IFN-β expression by binding to PRD II and I/III elements, respectively, which are nested within the enhancer of IFN-β gene [86]. Transcriptomic analysis of cGAMP-activated THP-1 cells showed that type I IFNs and ISGs, such as *IFNB1*, *IFIT1* and *CXCL10*, are among the most potently upregulated genes (> 50-fold above baseline) [89]. In total, more than 119 IFN genes are upregulated by NF-κB and IRF3 [89]. STING can catalyze TRAF3-dependent noncanonical activation of NF-κB, which involves the stabilization of NF-κB-inducing kinase (NIK) following TRAF3 degradation signaling [85, 90]. The non-canonical NF-κB pathway appears to have multiple roles in innate and adaptive immune responses [91].

Type I IFN production depends on the length of the dsDNA when present in low concentrations [27]. In PMA-differentiated THP-1 cells, longer DNA molecules induce greater cGAMP production, STING activation, TBK1 phosphorylation and type I IFN production [27]. Even though type I IFN production plateaus at higher DNA doses, cGAMP production, and the activation of STING and TBK1 positively correlate with DNA length, indicating that IFN production mediated by cGAS-STING saturates downstream of TBK1 [27]. This higher sensitivity to longer dsDNA is postulated to provide a preferential protective innate immune response against exogenous microbial DNA—which tends to be longer—while retaining tolerance to small DNA fragments, which are more likely to arise from innate nuclear DNA during mitosis [27].

The downstream effects of STING signaling seem to be downregulated by the inhibition of anaplastic lymphoma kinase (ALK), epidermal growth factor receptor (EGFR) and AKT. Inhibition of the ALK-EGFR-AKT pathway, *via* genetic disruption of ALK expression and pharmacological downregulation of ALK activity, attenuated cGAMP-induced ALK-EGFR interaction, as well as the phosphorylation of ALK-EGFR-AKT and components of the STING signaling pathway, TBK1, IRF3 and p65 [92]. IFN-β expression was similarly downregulated. However, ALK was not found to interact directly with cGAS, TBK1, or STING through immunoprecipitation analysis [92]. Regardless, because ALK fusion genes are associated with several types of cancer and these findings suggest a possible role for ALK in cGAS-STING signaling, the relationship between ALK-EGFR-AKT and components of the STING pathway ought to be thoroughly examined with further studies. STING is proposed to act as a hub to elicit differential downstream transcriptional responses depending on its mode of activation. Whereas cytosolic DNA triggers cGAMP production, STING translocation and activation *via* phosphorylation and palmitoylation, activation of STING following genotoxic DNA damage occurs *via* a different pathway. Etoposide-induced DNA damage activates STING in a cGAS- and cGAMP-independent manner *via* the serine/threonine kinase ataxia telangiectasia mutated (ATM), PARP1, p53 and IFI16, which then induces IFN production *via* TRAF6-mediated NF-κB activation [93].

### Mechanisms of cell death following cGAS-STING activation

Aside from its role in stimulating type I IFN production, cGAS-STING activity can also induce cell death through inflammasome activation, Beclin 1 (BECN1)-mediated autophagy, and Bcl-2 inhibition. For example, in human myeloid cells, upon the detection of cytosolic DNA, cGAS-STING causes the release of lysosomal hydrolases into the cytoplasm, leading to the degradation of cytoplasmic proteins and triggering lysosomal cell death (LCD) [94, 95]. LCD-mediated cell membrane disruption also facilitates K^+^ and Ca^2+^ movement, which induces the downstream NLRP3 inflammasome [95, 96]. Inflammasome-mediated pyroptosis then overtakes the LCD reaction by the more rapid action of active caspase-1 and its downstream lytic effects [95, 97]. When caspases are suppressed, however, BMDMs underwent STING-dependent, TNF-mediated necroptosis instead [98].

In previous studies, the treatment of murine splenic T cells with the small-molecule STING agonist, 10-carboxymethyl-9-acridanone (CMA), resulted in the expected TBK and NF-κB phosphorylation. However, instead of type I IFN production, the cells underwent apoptosis, with caspase-3 and -7 activation and positive Annexin V staining [99]. Consistent with this, others found that caspase-3/7 activation led to a muted IFN response as part of an immunologically silent form of cell death, as compared to necroptosis, which involves mitochondrial damage, mtDNA release, cGAS activity and IFN production [100]. The accelerated apoptosis in these murine cells involved IRF3-dependent upregulation of BH3-only proteins—phorbol-12-myristate-13-acetate-induced protein 1 (PMAIP1; NOXA), BCL2 binding component 3 (PUMA), BCL2 like 11 (BCL2L11; BIM) and BCL2-associated agonist of cell death (BAD)—as well as activation of the apoptosis regulator BCL2-associated protein-X (BAX), which neutralizes the effect of the anti-apoptotic protein BCL2 [99, 101]. These BH3-only proteins initiate apoptosis by inducing BAX-dependent mitochondrial outer membrane permeability (MOMP), which leads to caspase 9-driven caspase-3/7 activation [102]. Mitotic cell death in HeLa and human BJ hTERT fibroblasts also involves the activated cGAS-STING-IRF3 pathway, which inhibits the Bcl-xL-dependent suppression of BAX activation and MOMP [66]. Even though mtDNA that is released as part of mitochondria-dependent cell death normally activates cGAS-STING and its downstream immunostimulatory response, this process is inhibited by caspase-3/7 activation [103]. From the above studies, cGAS-STING is clearly capable of mediating both immunologically silent apoptosis and immune-activating necroptosis, but how these two forms of cell death are balanced remains unclear.

STING-mediated cell death is cell-type dependent. Unlike primary T cells which underwent apoptosis, primary MEFs, bone marrow-derived dendritic cells (BMDCs) and macrophages (BMDMs) were resistant to apoptosis when treated with CMA [99]. In both normal and malignant B cells, but not in MEFs, 3′-3′-cGAMP-activated STING leads to activation of Annexin V and caspases 3, 7, and 9 [104]. This cell type-dependent apoptotic response is due to a higher basal expression level and less efficient degradation of STING in B and T cells, leading to accumulation of STING upon activation, with a resultant exaggerated apoptotic response [99, 104].

The Beclin 1 (BECN1)-mediated autophagy pathway can also be activated by cGAS. When bound to DNA, cGAS sequesters BECN1 from Rubicon, which negatively regulates phosphatidylinositol-3 kinase catalytic subunit 3 (PIK3C3), a pro-autophagy protein. The dissociation of BECN1 derepresses PIK3C3, leading to downstream autophagosome maturation and autophagy induction [105]. Noting also that cGAMP activates STING-dependent autophagy, Liang *et al*. postulated that this allowed cells to transition from STING-mediated type I IFN production to autophagy during the course of assault by exogenous DNA [105]. In the same vein, Gui *et al*. showed recently that, upon binding cGAMP, STING translocates to the ER-Golgi intermediate compartment (ERGIC) and the Golgi where STING-containing ERGIC serves as a membrane source for LC3 lipidation, a key step in autophagosome formation [106]. cGAMP induces LC3 lipidation in a WIPI2- and ATG5-dependent but ULK- and VPS34-beclin kinase-independent manner [106]. Because STING from the sea anemone *Nematostella vectensis* induces autophagy but not IFN when stimulated by cGAMP, the authors postulated that autophagy induction is a primordial function of the cGAS-STING pathway [106]. Autophagy is also triggered in human lung fibroblasts and mammary epithelial cells experiencing telomeric crisis *via* cGAS-STING activation following the release of cytoplasmic DNA from micronuclei and cytoplasmic chromatin fragments [107].

## cGAS-STING ACTIVATION IS ASSOCIATED WITH CELLULAR SENESCENCE FOLLOWING DNA DAMAGE

Senescence is an intrinsic mechanism to persuade damaged or stressed cells to enter into permanent cell cycle arrest [108]. Yet, during senescence, some cells will still secrete inflammatory mediators *via* the cGAS-STING pathway, and are referred to as having a senescence-associated secretory phenotype (SASP). Indeed, cGAS-KO and STING-KO MEFs have higher proliferation rates following serial passaging as compared with WT MEFs, and this is accompanied by lower levels of senescence-associated β-galactosidase and cell-cycle inhibitors *Cdkn2a* (p16^INK4a^) and *Cdkn2b* (p15) in the knock-out cells [52]. Various other SASP genes were abundantly expressed in serially passaged WT MEFs, whereas genes that mediate mitotic cell division were downregulated [52]. These findings were reproduced in other studies [1, 2, 109], and collectively show that cGAS-STING is essential for the expression of SASP genes.

Besides its involvement in senescence *via* SASP gene regulation, cGAS-STING signaling is also implicated in senescence maintenance. In cells with established senescence, cGAS inactivation leads to a significant downregulation of SASP genes although senescence was not reversed [2]. In hepatic stellate cells from senescent STING-null mice, senescence markers are significantly reduced [109]. These observations corroborate previous findings showing that IFNβ is capable of inducing senescence by triggering ROS production [110], thus suggestive of a possible mechanism of senescence maintenance mediated by cGAS-STING. Activation of the cGAS-STING pathway is also associated with the presence of various inducers of senescence, including oxidation, ionizing radiation, cell cycle inhibition (CDK4 inhibitors, such as palbociclib), and DNA damage (cytotoxic agents, such as etoposide) [1, 52]. Furthermore, both cGAS and STING KO mice exposed to whole body radiation show reduced senescence. The expression levels of ISGs, SASP proteins (IL-6, Cxcl10, IL-1α) and cell-cycle inhibitors (*Cdkn2a*) in cGAS/STING-null mice were significantly attenuated as compared with WT mice [2, 52]. Several months post-irradiation, WT mice, but not STING null mice, exhibited massive hair greying [2].

Taken together, the cGAS-STING pathway confers protection against the proliferation of aberrant cells through a multi-pronged mechanism that imposes permanent cell cycle arrest to inhibit the proliferation of damaged cells, and recruits immune cells to clear the senesced cells. The significance of such a signaling pathway is especially critical in the context of immune-mediated monitoring and clearance of cancerous cells to thwart tumor development.

## cGAS REGULATES DNA DAMAGE-INDUCIBLE INFLAMMATORY RESPONSE

Aside from the cellular responses to cytosolic DNA described in previous sections, the cGAS-STING pathway will also activate myeloid cells and the adaptive immune system by associating with DNA released from apoptotic cells. There is huge variability in the responses of myeloid cells to cGAS-STING activation, with CD11b^+^ DCs the major group that ingests DNA to incite an inflammatory response. Comparatively, splenic marginal zone CD19^+^ DCs seem predisposed to inducing regulatory responses *via* the immunomodulatory enzyme indoleamine-2,3-dioxygenase (IDO) on sensing DNA [111]. CD8α^+^ DCs and CD169^+^ macrophages, on the other hand, do not react to DNA ingestion [111]. How these responses are connected is unclear but may be linked to the route of DNA uptake, the cell type, or the duration of DNA exposure.

Normal apoptotic cells do not accumulate cytosolic DNA: genomic DNA is promptly degraded by caspase-activated DNase, which prevents an immune response, while other DNA is degraded by lysosomal DNase II following phagocytosis [112, 113]. In the tumor microenvironment (TME), however, tumor nuclear DNA is accumulated and induces cGAS-STING signaling. Indeed, studies have reported the constitutive presence of cytosolic DNA in tumor cell lines such as YAC-1, EμM1, and PC-3 [3, 68, 114]. Stressful conditions, such as hypoxia, genomic instability, DNA damage or nutritional restriction, can trigger tumor cell apoptosis, resulting in the release of DNA into the extracellular space [115]. For example, topotecan-induced DNA damage within E0771 breast cancer cells causes the exosomal release of short (< 2 kbp) DNA fragments that are resistant to DNase treatment [116]. Cytosolic DNA associates with the late endosomal marker CD63, which can be recycled to the cell surface, perhaps suggestive of a route by which tumor cell cytosolic DNA is released into the extracellular compartment [3].

Phagocytosis of extracellular tumor DNA by intra-tumoral DCs triggers the activation of the cGAS-STING pathway [117–119]. DNA can leak into the cytoplasm of DCs in a manner similar to the phagosomal escape of proteins, which are eventually processed for presentation on MHC Class I molecules [120]. The direct uptake of free extracellular DNA by DCs is less plausible because of the need for cell–cell contact between tumor cells and DCs for IFNβ production and DC cross-priming activity [121]. DCs can also be activated to produce IFNβ by dsDNA exported in exosomes from irradiated tumor cells [122]. It is proposed that ROS production by tumor cells after radiation may stabilize the DNA by resisting degradation by Trex1, thereby facilitating efficient uptake by DCs [121]. CD11b^+^ macrophages and monocytes can also secrete IFNβ, Cxcl10, and TNFα in response to cytosolic DNA and cGAMP in engulfed cells, as well as intratumoral CDN [28, 123].

**Figure 2:**
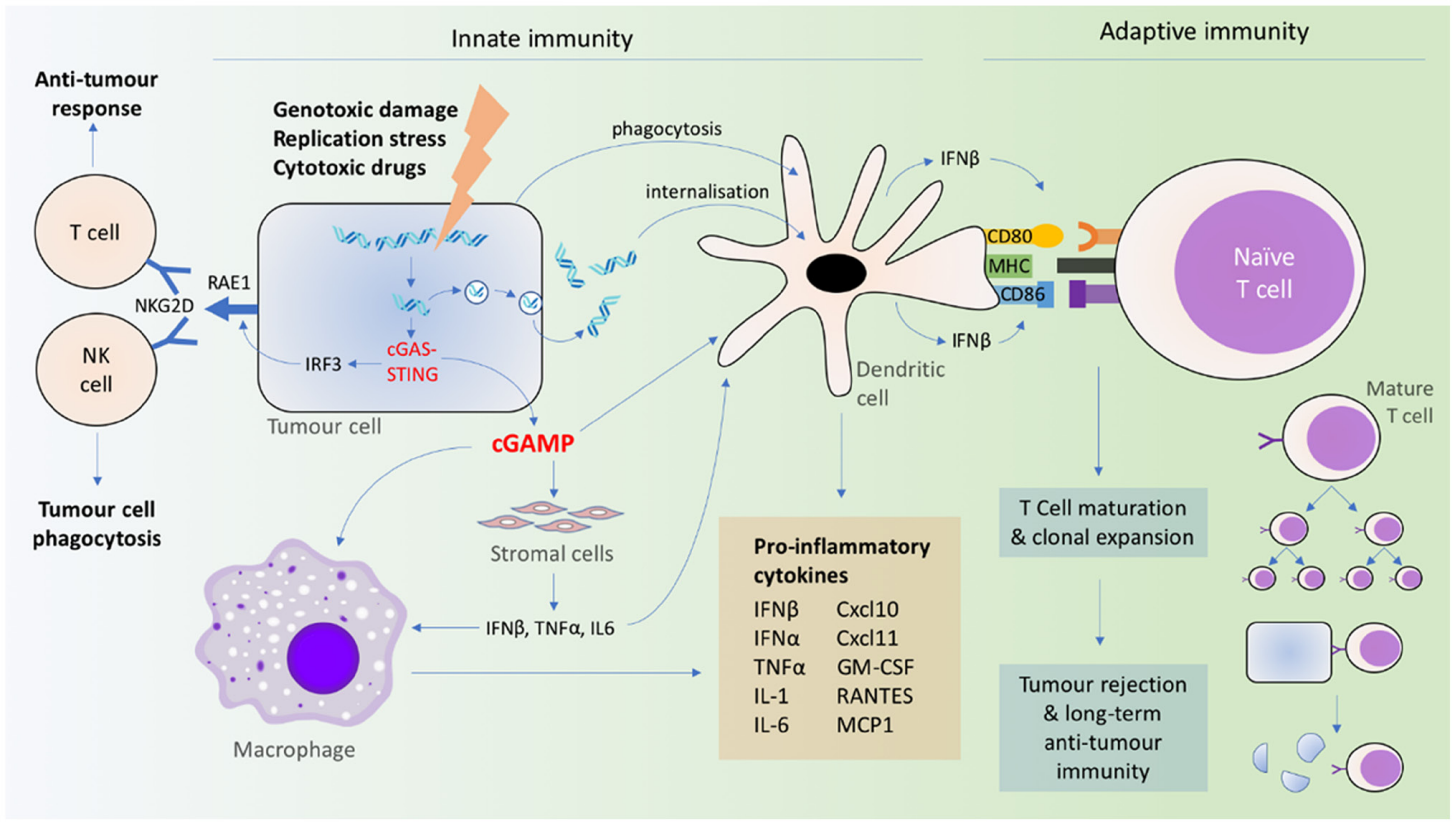
Coordination of innate and adaptive immune signaling resulting from cGAS-STING activation.

Cytosolic DNA resulting from DNA damage leads to the STING-IRF3-dependent expression of retinoic acid early transcript (RAE1) isoforms in murine B cell lymphoma cells [114]. RAE1 acts as a ligand of NKG2D on NK cells and certain T cells to facilitate tumor immunosurveillance [124] (Figure 2). Interestingly, NK cells, lymphocytes and macrophages are all important mediators of anti-tumor immunity downstream of cGAS-STING activation, depending on the immunogenicity of the specific tumor [125, 126].

Although the transfer of dsDNA from tumor to immune cells is a well-recognized mechanism for the activation of cGAS-STING, recent evidence suggests that transferred cGAMP has a role to play as well. Tumoral cGAMP was observed to transfer *via* gap junctions to astrocytes and activate astrocyte STING and IFN production, and the transmembrane transporter SLC19A1 has also been implicated in cellular uptake of CDNs [79, 82, 83]. cGAMP produced by constitutively active cGAS in tumor cells can trigger CD11b^+^ immune cells within the TME resulting in NK cell-dependent tumor rejection [125], and also stimulate STING-dependent type I IFN production in DCs and macrophages [126] (Figure 2). These findings support the role of released cGAMP as a mechanism for local immune cell activation.

The activation of the cGAS-STING pathway by both exported tumor dsDNA and cGAMP can partly be explained by the variable expression of cGAS and STING across different tumor cell types. It was found that a majority of human ovarian cancer cells lack either cGAS, STING, or both [127]. STING expression or signaling is similarly impaired in melanoma, colorectal and gastric cancer cells [128–130]. While the implications of cGAS/STING deficiency in cancer cells on immune clearance were not explored in these studies, it is possible that under such circumstances, cGAS-STING signaling is taken over by stromal or infiltrating immune cell cGAS/STING *via* the other previously described mechanisms (direct uptake of tumor dsDNA, cGAMP, or phagocytosis of tumor cells). Another paper has found that tumoral cGAS and host STING expression, specifically, are critical for an NK cell-mediated tumor rejection response [125], suggesting a complex interplay between both host and tumoral cGAS-STING signaling in mediating the downstream anti-tumor immune response.

cGAS-STING activation within the TME leads to an acute decrease in the proportion of tumor-infiltrating lymphocytes and an increase in tumor-infiltrating CD11b^+^ F4/80^+^ macrophages and CD11b^+^ F4/80^-^ neutrophils [123]. Mature CD11b^+^ Ly6C^+^ F4/80^+^ MHC Class II^+^ macrophages transiently migrate into the TME upon STING activation [131], resulting in the expression of innate cytokines, including IFNα, IFNβ, GM-CSF, IL-1, TNFα, MCP-1, and RANTES in DCs and macrophages [119, 123, 132, 133]. TNFα—predominantly produced by bone marrow-derived cells—mediates downstream anti-tumor immunity and tumor cell necrosis [123]. Prolonged cGAS-STING activation within the TME (> 5 h) causes cytokine levels to remain high within the tumoral and tumor-draining lymph nodes (TDLN), and it is unclear whether this is due to direct or indirect STING activity [123]. The recruited intratumoral macrophages demonstrated phagocytic function and a cytokine profile overlapping with that of the pro-inflammatory M1 type macrophages [131].

Cytosolic DNA is akin to a damage-associated molecular pattern, which is required for the activation of antigen presenting cells (APCs) [134]. In addition, direct phagocytosis of damaged cells induces DC activation, regardless of the cause of cell death [119]. Interestingly, APC activation is not limited to direct cGAS-STING activation in APCs, but can be triggered by inflammatory cytokines of neighboring STING-activated stromal cells [123] (Figure 2). In addition, neither bone marrow-derived nor stromal cells alone are sufficient in STING-mediated tumor clearance [123], once again pointing to a network involving complex interactions among tumor, stromal, and immune cells.

cGAS-STING activation coordinates not only a rapid innate immune response involving macrophages, monocytes, and granulocytes in the TME, but also the adaptive immune system involving lymphocytes and the release of type I IFN from DCs [121, 135] (Figure 2). Type I IFN unites the innate and adaptive immune response systems, in particular, by stimulating DCs [119, 136, 137]. This, in turn, primes tumor antigen-specific CD8^+^ T cells [119, 121, 136–138]. In these DCs, cGAS-STING (activated by dsDNA or cGAMP) triggers the production of IFN-β, cytokines (IL-12, IL-6, TNF-α), chemokines (CXCL9), and other co-stimulatory factors (CD86 and CD40) for T cell activation and clonal expansion in a dose-dependent manner [118, 121, 135, 139]. DC-produced IFNβ upregulates the self-expression of CD40, CD80, CD86, and MHC II [140], which further activates CD8^+^ T cells (Figure 2). In contrast, the direct sensing of type I IFN in CD8^+^ T cells, NK cells, granulocytes and macrophages are not required in tumor rejection [119, 137]. The process is also independent of TLRs [118], AIM2, DAI, RNA polymerase III, or high mobility group box (HMGB) proteins [139], suggesting the primacy of the cGAS-STING mechanism in mediating the anti-tumor immune response. The cGAS-STING pathway can also be activated in T cells in a T cell receptor-independent manner, and simultaneous STING agonism and T cell receptor activation have synergistic effects on downstream signaling [101].

The TME is usually highly immunosuppressed due to the secretion of tumor-derived growth factors (VEGF, TGFβ) [134], or macrophage-derived IL-10 [141], with significant infiltration of myeloid-derived suppressor cells (MDSCs) and tumor-associated macrophages (TAMs) [142]. The TME is infiltrated with CD8^+^ T cells, which have been linked to the recruitment of Foxp3^+^ regulatory T cells (Tregs) *via* CCR4-binding chemokines, as well as IFN-γ-induced IDO and PD-L1 expression, which are recognized immunosuppressive factors [143]. However, the activation of cGAS-STING can downregulate this immunosuppressed phenotype. For example, STING can suppress MDSC induction by enhancing SOCS1 expression and inhibiting STAT3, thereby reducing the production of granulocyte-macrophage colony-stimulating factor (GM-CSF) and IL-6, which are critical for MDSC differentiation [144]. Activation of STING also remodels the TME by reducing the proportion of infiltrating CD4^+^Foxp3^+^ Tregs [145] and promoting the polarization of M2-type (reparative) macrophages, which are anti-inflammatory and pro-tumorigenic, to M1-type macrophages, which are pro-inflammatory [146, 147]. This macrophage polarization is also associated with an increase in CXCL10 and IFNβ [148]. In the absence of cGAS (i.e., cGAS-deficient macrophages), the M2-type phenotype is maintained at sites of inflammation, with higher levels of fibronectin (M2 marker) and lower expression of IL-6 in response to IFNγ stimulation [148].

## cGAS-MEDIATED INFLAMMATION IN CANCER

STING has a vital role in the recruitment of immune cells to the tumor microenvironment, and has beneficial anti-tumoral effects. Indeed, reduced cGAS and STING expression are associated with poorer survival of patients with lung adenocarcinoma [1] and invasive breast ductal carcinoma [4]; albeit, there is no discernible association among patients with lung squamous cell carcinoma or ovarian cancer [1]. In both early- and late-stage gastric cancer, low intratumoral STING expression is associated with poorer survival. Using RNA-Seq analysis across several cancer genome databases, it was shown that cGAS and STING were specifically associated with an increase in pro-inflammatory gene expression in human cancers [2]. An important caveat is that intratumoral cGAS-STING expression, as determined *via* bulk RNA-Seq in most of these studies, comprises expressed cGAS and STING from both host (stromal and immune cells) and cancer cells. Therefore, the relationship between tumoral STING expression and cancer prognosis can be confounded by the infiltration of immune cells, which is included in the total expression of STING from the tumor. From a clinical standpoint, however, it might simply suffice to evaluate the overall STING expression as it has been found that combining STING expression with TNM staging results in a significant improvement in the sensitivity and specificity of predicting survival among these gastric cancer patients as compared with either model alone [130].

In cGAS/STING-deficient mice, the introduction of oncogenic NRasV12 into hepatocytes reduces the expression of SASP factors (IL-1α, IL-1β, IL-6, and Cxcl10), p21 and immune cell markers. At an early stage after oncogene delivery (12 days), WT mice showed significant immune-mediated clearance of Nras-positive cells as compared with the cGAS/STING-deficient mice [2, 52]. Much later (8 months), whereas there were no intrahepatic Nras-positive tumors in the WT mice, tumors were still present in STING-null mice [2]. In another glioma mouse model, STING-deficient mice experienced reduced survival, with a lower expression of type I IFNs in the glioma-bearing cerebral hemisphere [149]. A loss of cGAS-STING can also render mice more susceptible than WT mice to developing colitis-associated colonic adenocarcinoma when subjected to treatment with DNA damage-inducing agents. The tumors from STING-deficient mice showed significant inflammatory cell infiltration and reduced expression of genes such as Cxcl13 and Ccr6 [150]. One of the STING-dependent genes, IL-18, may hold the key to the mechanism of these changes: IL-18, produced in response to DNA damage, inhibits IL-22BP, thereby derepressing IL-22, a cytokine involved in tissue regeneration, repair, and proliferation [150, 151]. IL-22 is normally regulated by secreted IL-22BP from CD11c^+^ DCs; if left unchecked, IL-22 can lead to tumorigenesis [151].

Others have shown that cGAS knockdown leads to reduced DNA damage, inhibited tumor growth, and inhibited malignant cell transformation, whereas cGAS overexpression enhances the malignant potential of tumor cells *in vitro* and *in vivo* [69]. Furthermore, studies have shown that carboplatin combined with the gap junction modulators meclofenamate and tonabersat, which block cGAMP transfer, can significantly control brain metastasis *in vivo* [79]. At present, there is one ongoing clinical trial (ClinicalTrials. gov, NCT02429570) studying the efficacy of meclofenamate monotherapy in the treatment of metastatic brain cancer, and the findings from this study will represent an important step towards advancing the treatment of metastases, which remains a dismal prognosis for patients.

### Pro-tumorigenic effects of cGAS-STING activation

Given that tumor cells frequently undergo necrosis, DNA damage and nuclear rupture—all of which would expose the cytoplasm to an abundance of genomic DNA—the question remains: why are tumor cells not critically inhibited and destroyed by cGAS-STING-mediated immunosurveillance? As it turns out, although the cGAS-STING pathway has clear anti-tumor benefits [i.e., stimulating immune cell-mediated clearance of (pre-) malignant cells], chronic cGAS-STING activation due to the persistence of cytosolic DNA causes the opposite effect, inflammation-mediated tumorigenesis. For example, inactivation of the BRCA2 gene causes impaired DNA repair and micronuclei accumulation, which activates the cGAS-STING pathway, resulting in cell cycle arrest, increased IFN signaling, and increased TNFα secretion, triggering cell death [152, 153]. However, in chronic BRCA2 inactivation and, consequently, chronic cGAS stimulation, not only are ISGs upregulated, but cell cycle progression is restored, which promotes the survival of mutated cells [153].

Compared with nascent or primary tumors, metastatic tumors are associated with chromosomal abnormalities such as aneuploidy and even polyploidy, which can arise from a mis-segregation of chromosomes during anaphase [56]. Due to the ubiquity of cytosolic DNA in chromosomally unstable cancer cells, cGAS-STING and the non-canonical NF-κB pathway were found to be activated. In fact, the increased expression of non-canonical NF-κB pathway target genes was associated with higher levels of chromosomal instability and poorer clinical prognosis and survival [56]. Further supporting the idea that the non-canonical pathway enhances cancer cell fitness is the observation that inactivating mutations in cGAS and STING are rarely found in breast or lung cancer cells [56]. On the other hand, increased expression of canonical NF-κB was associated with improved prognosis [56].

While cGAS-STING indisputably plays an important role in mediating the anti-tumor immunity, the above findings showing that chronic cGAS-STING activation and induction of the STING-dependent non-canonical NF-κB pathway seem to be pro-tumorigenic warrant further studies to clearly delineate the mechanisms driving these dichotomous outcomes.

## EVASION OF cGAS-STING MEDIATED ANTI-TUMOR IMMUNITY IN CANCER

Based on the non-redundancy of STING in response to cytosolic DNA sensing, as evidenced by the lack of other cellular receptors for cGAMP [89], cancer cells stand to gain by evading STING-mediated innate immune responses. Indeed, there is data to show that certain malignant cells can circumvent the cGAS-STING-mediated immune response by either targeting cGAS-STING expression, or by suppressing their function(s). Yet, other evidence points to tumorigenesis resulting from both chronic, elevated STING activation as well as STING-deficient situations.

### Transcriptional regulation of cGAS and STING expression

Compared with normal gastric epithelia, STING expression is significantly reduced in surgically resected samples of gastric cancer [130]. In gastric cancer cell lines, the delivery of dsDNA or cGAMP can reduce STING expression, and STING knockdown leads to increased growth with enhanced migratory and invasive potential [130]. These findings suggest that low STING expression may diminish anti-tumor immunity.

cGAS expression is downregulated under hypoxic cellular conditions, which is common in the TME. Within the hypoxic TME, HIF-1α induces the production of the hypoxia-responsive miRNAs, miR-25 and miR-93, to establish an immunosuppressive microenvironment. This is partly brought about by downregulating the function of nuclear receptor coactivator 3 (NCOA3), which is necessary for maintaining basal cGAS levels [4]. Under hypoxic conditions, cells undergo mitophagy and mitochondrial fragmentation, releasing mtDNA into the cytosol [154]. However, cGAS expression and type I IFN production are simultaneously muted by miR-25/93, leading to the suppression of CD8^+^ T cell-mediated anti-tumor immunity and the establishment of an immunosuppressive environment with Treg and MDSC recruitment [4]. miR-25/93 have recently emerged as oncogenic miRNAs, and this mechanism highlights its role in tumorigenesis [155]. In fact, miR-25/93 expression is a negative prognostic marker for hypoxic tumors, such as brain, colon, and breast tumors, with higher levels of miR-25/93 correlating with reduced survival in patients with invasive breast ductal carcinoma [4].

dsDNA signaling can be dysregulated at multiple points in the cGAS-STING activation cascade, from the level of gene transcription to the engagement of downstream mediators. Interestingly, significant cGAS or STING mutations and deletion events are infrequent [128], with a prevalence of <1% [156]. However, there is a higher frequency of cGAS/STING gene promoter silencing by hypermethylation in cancer cells as compared with WT cells [156]. Yet, given that these hypermethylation events cannot be fully rescued by demethylating agents, it is possible that other mechanisms of gene suppression are at play, including histone modification and miRNA regulation [128, 129]. Additionally, the ability of STING to activate downstream targets, such as IRF3 or NF-κB, can also be compromised in cancer cells, but this occurs *via* unknown mechanisms [129].

Some cancer cells lengthen their telomeres through the ALT (alternative lengthening of telomeres) pathway, which involves the addition of telomeric repeats by HR instead of telomerase activity [157]. STING suppression is implicated in this pathway [158]. Extrachromosomal telomere repeat (ECTR) dsDNA, a by-product of the ALT pathway [159], is unique to cancer cells. Induction of ECTR dsDNA in human fibroblasts can activate cGAS-STING-mediated IFNβ production and induce senescence. However, this does not occur in ALT cancer cell lines [158], despite the abundance of ECTR. Instead, STING (and cGAS, in some cases) expression is abolished in ALT cancer cell lines and ALT transformed cells, suggesting that the loss of cGAS-STING is requisite for the progression of ALT cancers [158]. Through ALT cancer cells, this study highlights that cGAS-STING can be downregulated not only through epigenetic transcriptional mechanisms, but also through post-transcriptional control [158].

Although the downregulation or silencing of cGAS-STING within tumor cells might limit host anti-tumor immunity, it is precisely this mechanism that also renders the cells more susceptible to viral oncolysis. This has been shown using a strain of the Herpes Simplex Virus (HSV) lacking the γ34.5 gene [128, 129]. Under these conditions, cGAS-STING signaling activity is inversely related to viral replication and cell death [128]. Thus, even though therapies that leverage cGAS-STING signaling to initiate an anti-tumor response might be less effective in these tumors, viral oncolysis can serve as a viable alternative.

In tumors with low antigenicity, such as Lewis lung carcinoma (LLC), cytosolic DNA sensing and STING activation within the normal host tissue lead to tumor growth rather than regression [160]. STING and type I IFNs induce the production of indoleamine 2,3-dioxygenase (IDO), which plays a role in immunoregulation. However, with enhanced LLC antigenicity host STING activation does not promote tumor growth or induce IDO production [160]. This finding suggests that the downstream effects of STING activation are highly dependent on tumor antigenicity. With reduced antigenicity, STING activation can induce tolerogenic immune responses and facilitate tumor progression; with enhanced antigenicity, STING activation can induce potent innate and adaptive anti-tumor immune responses *via* APCs and CD8^+^ T cells.

### Suppression of cGAS-STING function

STING suppression offers another way in which cancer cells can evade cGAS-STING signaling. In HER2-mutated cancer cells, HER2 associates with STING and recruits AKT1 to phosphorylate TBK1, the downstream signaling target of STING, thereby preventing STING-TBK1 and TBK1-IRF3 interactions. Suppressing cGAS-STING signaling downregulates cytokine and senescence expression and inhibits apoptosis, protecting cancer cells from the host anti-tumor immunity [161].

## HARNESSING THE cGAS-STING PATHWAY TO TREAT MALIGNANCIES

cGAS-STING signaling undoubtedly plays an important, centralized role in the immune-mediated clearance of malignant cells, and the positive correlation between cGAS-STING pathway integrity and cancer survival positions cGAS-STING as a candidate drug target for pharmacological intervention against cancers and other self-DNA-induced inflammatory conditions. Despite the tumorigenic role of chronic cGAS-STING activation, its role in intra-tumoral immune sensing may offer significant therapeutic potential. Indeed, many studies have endeavored to harness the potency of cGAS-mediated immune response in cancer treatment.

### Monotherapy of cGAS-STING agonists in the treatment of cancer

Based on the idea that cGAS-STING activation can induce a potent immune-mediated response against tumor cells, several studies have sought to explore the possibility of using STING agonists as adjuvants or chemotherapeutic agents to enhance immunotherapy. The intratumoral administration of CDNs has drawn particular attention. For example, treating mice bearing gliomas with c-di-GMP induced STING-dependent ISG expression, increased TILs and an inhibition of tumor growth. This anti-tumor effect was further enhanced by combining c-di-GMP treatment with tumor vaccines [149]. Similar tumor growth attenuation and prolonged survival have been shown in mouse models of 4T1 breast cancer, mSCC1 squamous cell carcinoma, CT26 colon cancer, C26 colon adenocarcinoma and B16F10 melanoma tumors treated with cGAMP [131, 140, 162]. Interestingly, treatment with CDNs is also capable of regressing distant tumors, known as the abscopal effect: Multiple murine studies have revealed that intratumoral injection of STING agonists can not only induce primary tumor regression, but also the regression of distant metastases or implanted tumors [133, 145, 162–164]. This effect is proposed to be mediated by the activation of host adaptive immunity in response to DNA damage, which in turn triggers an anti-tumor response; this is as opposed to a simply systemic distribution of the injected cGAMP [58, 162].

Robust STING expression in the host is a critical factor determining the efficacy of STING agonists as anti-cancer agents. Treatment of STING^−/−^ mice carrying STING-expressing tumors with the STING agonist ADU-S100 elicited a transient growth impairment of the primary and secondary tumors, whereas STING^+/+^ mice carrying STING-KO tumors showed complete clearance of the primary tumor along with delayed secondary tumor growth [145]. In T cells from B6 mice, treatment with the STING agonist 5,6-dimethylxanthenone-4-acetic acid (DMXAA, Vadimezan, ASA404) induced the expression of STING-target ISGs, IFNβ, IFNγ, TNFα and IL-6 [101, 165]. Such encouraging preclinical data showed further potential in phase I/II clinical trials, but unfortunately failed in phase III trials; this failure may be due to structural differences between murine and human STING [166–168]. The route of STING administration is another factor that may have an impact on therapeutic efficacy. Intratumoral injection of the STING agonist cGAMP in mice resulted in potent inflammation and tumor regression with no adverse off-target effects. In contrast, the intraperitoneal injection of DMXAA produced signs of sterile shock in mice [98, 132].

Of note, treatment with STING agonists can circumvent immune evasion of disseminated cancer cells. In a model of acute myeloid leukemia (AML), in which leukemia antigen-specific CD8^+^ T cells become inactivated and host type I IFN secretion becomes impaired, direct STING activation by intravenous administration of DMXAA or CMA can ectopically induce type I IFN release to activate host DCs and T cells and maintain immunological memory, thereby improving the survival of the leukemic mice [165, 169].

Chemically synthesized CDNs have been employed for anti-tumor therapy. For example, dithio-(Rp, Rp)-[cyclic[A (2’,5′) pA (3′,5′) p]] (ML-RR-CDA) has been shown to be a potential STING agonist in the clinic [133]. Relative to endogenous cGAMP, this synthetic CDN, with its 2’-3′ mixed linkage bridge, has a higher binding affinity for human STING [170], can induce greater secretion of IFNα, and offered persistent and specific T cell-mediated immunological memory [133, 171]. In another murine B cell lymphoma model, the synthetic nucleotide STING agonist cyclic dithio-modified diadenosine (ADU-S100) led to host STING-dependent clearance of the tumor, with intratumoral injection resulting in complete local tumor clearance and scar formation [145]. And finally, another small-molecule non-nucleotide-based STING agonist was shown to induce a robust adaptive CD8^+^ T cell-mediated immune response, inhibition of *in vivo* colon tumor growth and improved survival when delivered intravenously [172].

Because of the role of cGAMP export in triggering the immune response, a new study sought to exploit ENPP1, the enzyme responsible for extracellular degradation of cGAMP, by developing a small molecule inhibitor, STF-1623 [173]. Administered systemically, STF-1623 delayed growth of implanted Panc02 tumor in mice [173].

### Modified delivery vehicles for CDN administration

CDNs, such as cGAMP, carry negative charges and are thus hydrophilic, which restricts their diffusion into the cytosol of target cells. Thus, to enhance the cellular internalization of cGAMP, a cationic liposomal delivery vehicle was developed. This delivery vehicle not only improved tumor volume control but also survival in mice bearing PD-L1-insensitive tumors (including triple-negative breast cancer and B16F10 melanoma) as compared with soluble cGAMP or anti-PD-L1 therapy [147]. Systemically delivered liposomal cGAMP along with anti-PD-1 and anti-CTLA4 treatment further shrunk the size of the pulmonary metastases of melanoma in mice, with persistent anti-tumor immunological memory [174].

Likewise, a biodegradable poly (beta-amino ester) nanoparticle-based CDN delivery system improved drug potency more than 100-fold as compared with free CDN administration *in vitro*, and improved CDN delivery and immune cell internalization [175]. When combined with anti-PD-1 antibody immunotherapy, CDN-nanoparticles controlled B16-F1 melanoma tumor growth *in vivo* with a similar efficacy to a much higher dose (10×) of free CDN [175].

### STING agonist vaccine adjuvants

Several studies have explored the utility of cGAMP/STING agonists as vaccine adjuvants. In a previous murine B cell lymphoma study, vaccination of mice with a STING agonist and anti-GITR yielded a CD8^+^ T cell response that was reactivated when the mice were re-exposed to A20 B-cell lymphoma cells [145]. Compared with other adjuvants, a vaccine combining irradiated GM-CSF-expressing tumor cells and CDNs demonstrated improved anti-tumor responses against B16 melanoma, CT26 colon carcinoma, SCCFVII squamous cell carcinoma and Panc02 pancreatic carcinoma *in vivo* [171]. Another group developed a different neoantigen-targeted vaccine by whole exome sequencing, RNA-Seq and in silico methods and similarly combined it with the STING adjuvant ADU-V16 to target Panc02 cancer cells [176]. The administration of these vaccines resulted in a STING- and type I IFN-dependent increase in tumor-infiltrating CD8^+^ T cells [171, 176]. Similarly, combining STING-based vaccines with anti-PD1 and agonist OX40 antibodies resulted in a better inhibition of tumor growth, as compared with the vaccine and anti-PD1 combination or control, by augmenting and prolonging the lifespan of the T cell response. In turn, this created a long-term anti-tumor immunological memory as well as near-complete eradication of the tumors [171, 176].

A vaccine-based therapy involving the injection of irradiated B16 OVA cells containing a 90-bp 5′-modified AT-rich dsDNA—thus a cGAS ligand—into mice with metastatic B16 melanoma resulted in prolonged survival as compared with the control [28]. Combined with anti-PD-1 antibodies, the survival rates were significantly improved, suggesting that cGAS-STING ligands could improve the anti-tumor activity of other established therapeutic agents and vice versa [28].

### Targeting cGAS-STING in combination with other anti-cancer therapies

Genetic pathways often act collaboratively, and this holds true for mechanisms related to cancer cell survival, with communication between avoidance and resistance pathways likely to contribute to cancer cell growth and metastasis. This is where a synthetic lethality approach can be effective, even against hitherto untreatable cancers. Synthetic lethality describes the process by which two or more oncogenic pathways are simultaneously targeted to achieve or enhance a therapeutic response [177–180]. Given that the cGAS-STING pathway underpins a highly fundamental immunological response of cells against the presence of cytosolic DNA, it stands to reason that the cGAS-STING pathway could conceptually be modulated in sync with chemotherapeutic and/or immunotherapeutic means to achieve better treatment responses.

### Combining cGAS-STING activation with conventional chemotherapy/radiotherapy

The primary aim of anti-cancer treatments—whether conventional chemotherapy, radiotherapy or immunotherapy—is to reduce tumor volume and release tumor-specific antigens to prime the immune system [181]. Several previous studies have shown that radiation therapy achieves its intended anti-tumor effects through cGAS-STING-dependent immune activation [58, 121, 122]. Additionally, radiotherapy typically causes an influx of IL-10-secreting immunosuppressive M2-phenotype macrophages [182], inhibits IL-12 production [183] and affects NF-κB activity [184], which can contribute to radiotherapy resistance. Because radiotherapy can induce anti-tumor T cell responses by inducing type I IFN production in a cGAS-STING-dependent manner [121], cancer cells can develop resistance to radiotherapy through a loss of cGAS-STING signaling [5, 28, 58]. Thus, combining radiotherapy with immunostimulatory CDNs could potentially sensitize tumors to anti-cancer therapy.

A dose-dependent synergistic effect of CDN co-administration with radiotherapy was observed in mice bearing pancreatic adenocarcinoma or MC38 colon adenocarcinoma [121, 163]. The tumor cells showed significant hemorrhagic necrosis due to a disruption in the neoangiogenic vasculature induced by TNFα [163]. Additionally, the immunosuppressive M2 macrophage phenotype, which normally arises as part of a wound-healing response to radiotherapy, was abrogated by STING activation although no proinflammatory anti-tumor M1 phenotype was induced [163]. The number of IFNγ-producing CD8^+^ T cells in tumor-draining lymph nodes (TDLNs) was also increased in a STING-dependent manner as compared with either therapy alone [121]. Whereas high-dose CDN monotherapy was sufficient to control tumor growth *via* innate immune responses, lower doses of CDN, coupled with radiotherapy, could stimulate the adaptive immune response involving CD8^+^ T cells [163]. This finding highlights the potential of combining radiotherapy with immunotherapy to overcome the immune suppression of the TME and augment current treatment protocols.

However, a study by Hou *et al*. raises concerns about therapeutic approaches that use CDNs that only target STING and fail to discriminate between canonical and non-canonical downstream NF-κB pathways. The authors showed that the concomitant activation of the canonical and non-canonical NF-κB pathways suppresses IFNβ production, and dampens the therapeutic effect of radiotherapy [185].

Synergistic drug effects mean that the required dose of each constituent drug is reduced, and this helps to attenuate side effects or other toxicities associated with the administration of high doses [186]. Because each drug targets a different part of the cancer [179, 187], it is possible that cGAS-STING agonists can maximize the downstream anti-tumor response while reducing the required dose of the conventional chemotherapy drug(s), and the potential side effects. For example, combining cGAMP with 5-fluorouracil significantly increases survival and alleviates the severity of chemotherapy-induced mucositis, as marked by intestinal atrophy, in mice with colon adenocarcinoma tumors [140].

Finally, poly (ADP-ribose) polymerase (PARP), a key factor regulating the repair of DNA strand breaks *via* non-homologous end joining and micro-homology end joining pathways, is another potential treatment target [179, 180]. PARP has drawn much attention after several reports showing its synergistic action alongside key controllers of HR, which sparked a surge of interest in the clinical applications of PARP inhibitors (PARPi) alongside agents that target DNA strand breaks, especially in patients with loss-of-function polymorphisms in BRCA genes [179, 180]. Interestingly, cGAS-STING is also implicated in the mechanism of action of PARPi used in the treatment of BRCA1 or BRCA2-positive breast and ovarian cancer–PARP inhibition results in the formation of cytosolic dsDNA, which activates cGAS-STING and leads to IFN- and T cell-mediated anti-tumor responses [188]. Similarly, the administration of topotecan, an inhibitor of topoisomerase I, in E0771 murine breast cancer cells leads to the release of immunogenic DNA that induced cGAS-STING-dependent signaling, thereby driving anti-tumor immunity [116]. However, high levels of topotecan can attenuate this immune response *via* the death of proliferating immune cells [189], prompting caution with regards to the optimization of dosages.

### Combining cGAS-STING activation with immune checkpoint blockade therapy

Numerous factors within the TME affect T cell anti-tumoral responses. For instance, MDSCs express Fas ligands, which bind to Fas receptors on TILs to induce apoptosis [190], and the interactions between PD-1 on T cells and PD-L1 on tumor cells lead very quickly to T cell exhaustion [191]. Therefore, STING agonists alone are not the answer to eliminating cancer cells. The solution to this issue might lie in their combination with immune checkpoint blockade (ICB) therapy, which has gained prominence in recent years and demonstrated immense potential for cancer therapy, with anti-PD-1, anti-CTLA4, anti-CD47 now widely in use as anti-cancer agents [192].

Consistent with the essential role of cGAS-STING signaling in innate immune sensing and priming T cell-mediated anti-tumor immunity, STING is needed to mediate the anti-tumor effects of ICB therapy. For example, STING-deficient mice treated with anti-CTLA4 and anti-PDL1 mAbs showed poorer B16 melanoma tumor control than WT mice [118]. cGAS-STING is indispensable in the therapeutic effect of PD-L1 [135] or CD47 [117, 193] blockade against B16F10 mouse melanoma and MC38 colon adenocarcinoma *in vivo*. Both ICB therapies achieved a reduction in tumor volumes by recruiting tumor-infiltrating CD8^+^ and CD4^+^ T cells [117, 135, 193].

Remarkably, combining ICB with CDNs can enhance the production of tumor antigen-specific CD8^+^ T cells, leading to improved tumor control. The ICB anti-tumor effect can be further enhanced in a dose-dependent manner by the intramuscular delivery of cGAMP at a distant site [135]. Similarly, intratumoral cGAMP injection results in a dose-dependent inhibition of subcutaneous B16 tumor growth when combined with anti-CTLA4 and anti-PD-1 mAb treatment [162]. Apart from cGAMP, tumor cells loaded with 5′-modified dsDNA, combined with anti-PD1 mAb, can also improve survival rates in B16 melanoma-bearing mice [28].

Ager *et al*. comprehensively studied the TME changes within TRAMP-C2 prostate tumors in response to c-di-GMP and anti-PD-1, anti-CTLA4 and anti-4-1BB combination therapy. TRAMP-C2 tumors predominantly contain myeloid cells (CD11b^+^F4/80^+^Gr-1^-^ TAMs and CD11b^+^Gr-1^+^ MDSCs), with T cells (CD8^+^, CD4^+^FoxP3^-^ effector T cells and CD4^+^FoxP3^+^ Tregs) making up 5% of all CD45^+^ cells [164]. In response to intratumoral c-di-GMP and checkpoint modulators, the proportion of CD8^+^ T cells to Tregs, MDSCs, and M2 macrophages increased through Treg antibody-dependent cellular cytotoxicity, and resulted in M2 to M1 macrophage repolarization [164]. Similarly, the co-administration of intratumoral ADU-S100 with OX40 agonist and PD-L1 antagonist resulted in the enhanced activation of HER-2-specific CD8^+^ T cells and clearance of tumor cells in immune-tolerant neu/N mice compared with ADU-S100 monotherapy, suggesting that ICBs can potentiate the anti-tumor effect of CDNs [194].

Overall, these studies support the synergistic actions of cGAS-STING activation and ICBs to attenuate immune tolerance in tumor cells and enhance the recruitment and priming of anti-tumor immune cells. Combination therapy consisting of cGAMP and ICBs thus represents a new paradigm in the management of tolerogenic cancers. In fact, the STING agonist ADU-S100 is currently being studied as monotherapy or in combination with anti-PD1 (spartalizumab; PDR001) for the treatment of advanced, treatment-refractory metastatic solid tumors/lymphomas in two separate phase 1 clinical trials (https://clinicaltrials.gov/, NCT02675439 and NCT03172936).

### Risks of inflammatory conditions with cGAS/STING-based cancer treatments

The inflammatory pathway mediated by cGAS-STING is capable of potent anti-tumor responses but can also tip the system into a state of inflammation. cGAS-mediated inflammation has been recognized to be at the root of a multitude of diseases, ranging from systemic rheumatological conditions, including rheumatoid arthritis (RA) and systemic lupus erythematosus (SLE), to organ-specific pathologies such as myocardial infarction (MI).

Activating STING mutations have been implicated in an autoimmune condition known as STING-associated vasculopathy with onset in infancy (SAVI), in which the mutant STING localizes to the Golgi within the fibroblasts of affected patients, which in turn results in the constitutive activation of IFN expression even in the absence of cGAMP stimulation [195]. Such augmented IFN expression results in a debilitating condition characterized by cutaneous vasculopathy, respiratory and systemic inflammation [196]. Other pathological modifications to the cGAS-STING pathway, such as mutations of the exonuclease Trex1, have been linked to several other autoimmune conditions, including Aicardi-Goutières syndrome (AGS), SLE, familial chilblain lupus and retinal vasculopathy with cerebral leukodystrophy (RVCL) [197–199]. These mutations impair the degradation of cytosolic DNA by Trex1, resulting in an overactive cGAS response that underlies the inflammatory phenotype in Trex1-null mice [70].

The connection between the anti-tumor response and autoimmunity highlights the dual nature of the cGAS-STING-mediated response. On one hand, cGAS-STING activating therapies can trigger an intended pro-inflammatory response to cancers, but it can potentially also initiate a cascade of autoimmunity and inflammation, which can be as devastating as the cancers these treatments were designed to circumvent. As an example, TNFα, which is a recognized anti-tumor cytokine and a component of cGAS-mediated anti-tumor response, is also a central driver of RA pathogenesis. TNFα is normally expressed by DCs and T cells in response to cGAS-STING activation, but in the context of RA, it stimulates the expression of cGAS which proceeds to potentiate the inflammatory response in RA synoviocytes [200]. Whether TNFα produced in antitumor responses interact with synoviocytes to cause RA is not known. Likewise, cGAS-stimulated inflammatory M1 macrophages are present in both the TME and in cardiac tissues after a myocardial infarction. Unlike the beneficial role they play in the TME, these macrophages suppress cardiac tissue repair and worsen survival in mice post-MI [148].

While studies have not demonstrated a direct link between cGAS agonists and autoimmune conditions, these examples underscore the interconnected nature of the cGAS-mediated anti-tumor pathways and inflammatory diseases, with significant implications for applying cGAS/STING-based anti-cancer treatments: (1) more studies are needed to identify the full spectrum of human diseases mediated by cGAS-STING inflammation; (2) future studies should evaluate the long term effects of cGAS-STING agonists not only on cancer remission, but also on the development of other chronic conditions; and (3) strategies should be developed to adequately mitigate these potential risks associated with cGAS/STING-based therapies.

## CONCLUDING REMARKS: cGAS-STING PATHWAY AND CANCER TREATMENT

Considering the differential expression of STING in various cell types, it is reasonable to question whether the use of STING ligands will be effective in the treatment of cancer. Fortunately, *in vivo*, STING expression in cancer cells is not as critical for STING ligand-induced anti-tumor immunity as STING expression in the tumor stroma [163].

Immunogenicity varies among different murine tumor models, and this in turn will lead to differing responses following STING ligation. For example, the murine MC38 colon adenocarcinoma, 4T1 metastatic breast cancer and CT26 colorectal carcinoma tumor models have higher proportions of tumor-infiltrating cells [201], and thus respond better to CDN treatment, and can initiate more effective adaptive responses, with less reliance on the innate mechanisms [123]. In comparison, weakly immunogenic tumors, such as the murine B16F10 melanoma model, rely on more innate immune mechanisms, with a greater focus on cytokines (e.g., TNFα) to bring about tumor cell death [123]. Similarly, among solid tumors, Hepa 1-6 hepatoma and LL/2 Lewis lung cancer cells do not respond to treatment with cGAMP, DMXAA, or CMA [104]. A consequence of the absence of adaptive immune priming could be the blunted ability to exert any anti-tumor effect on distant tumor sites and prevent tumor recurrence. While still debated, this hypothesis would mean that cGAS-STING ligands should not be indiscriminately employed for the treatment of all cancers.

Additionally, amidst the excitement surrounding the plethora of anti-cancer therapies capitalizing on stimulating the cGAS-STING pathway, it is critically important to consider the fine line separating anti-tumor immunity from autoimmunity and to thoroughly and cautiously evaluate all treatment options to better understand the risks of these treatment modalities.

This review summarizes the critical role of the cGAS-STING pathway in mediating the autophagic, innate, and adaptive immune responses to cytosolic DNA arising from tumor cells. While there is great potential for using cGAS-STING agonists as anti-cancer agents, their use as clinical therapeutics should proceed cautiously due to the risk of tumorigenesis associated with chronic cGAS-STING activation and non-canonical downstream signaling. Defining the immune profile of tumors and elucidating the pathways controlling the anti- and pro-tumor effects of cGAS-STING activation will pave the way forward for its implementation in anti-cancer therapy.

## Abbreviations

cGAS: cyclic GMP-AMP synthase
STING: Stimulator of interferon genes
IFN: interferon
PRR: pattern recognition receptor
DAMP: danger-associated molecular pattern
PAMP: pathogen-associated molecular pattern
TLR: Toll-like receptor
NOD: nucleotide-binding oligomerization domain
RIG-I: retinoid acid-inducible gene I
CDN: cyclic dinucleotide
cGAMP: cyclic GMP-AMP
dsDNA: double-stranded DNA
dsRNA: double-stranded RNA
ssDNA: single-stranded DNA
ssRNA: single-stranded RNA
PBMC: peripheral blood mononuclear cells
ER: endoplasmic reticulum
TBK1: TANK-binding kinase 1
NAK: NF-κB-activating kinase
IRF3: interferon regulatory factor 3
TRAF6: TNF-receptor associated factor 6
ISG: IFN-stimulated genes
NIK: NF-κB-inducing kinase
ALK: anaplastic lymphoma kinase
ENPP1: ecto-nucleotide pyrophosphate phosphodiesterase 1
DSB: double-stranded break
CCF: cytoplasmic chromatin fragment
mtDNA: mitochondrial DNA
ROS: reactive oxygen species
NET: neutrophil extracellular trap
LCD: lysosomal cell death
ERGIC: ER-Golgi intermediate compartment
CMA: 10-carboxymethyl-9-acridanone
PMAIP1: phorbol-12-myristate-13-acetate-induced protein 1
BCL2L11: BCL2 like 11
BAD: BCL2-associated agonist of cell death
BAX: BCL2-associated protein X
MOMP: mitochondrial outer membrane permeability
BECN1: Beclin 1
VEGF: vascular endothelial growth factor
PIK3C3: phosphatidylinositol-3 kinase catalytic subunit 3
BMDC: bone marrow derived dendritic cell
BMDM: bone marrow derived dendritic macrophage
SASP: senescence associated secretory phenotype
TME: tumor microenvironment
DC: dendritic cell
RAE1: retinoic acid early transcript 1
TDLN: tumor-draining lymph node
APC: antigen presenting cell
HMGB: high mobility group box
TGFβ: transforming growth factor beta
MDSC: myeloid derived suppressor cell
TAM: tumor associated macrophage
Tregs: regulatory T cells
GM-CSF: granulocyte-macrophage colony-stimulating factor
TNM: Tumor-Node-Metastasis
BRCA: breast cancer associated
EMT: epithelial-to-mesenchymal transition
DFMS: distant metastasis-free survival
RFC1: Reduced Folate Carrier 1
HSV: Herpes Simplex Virus
LLC: Lewis lung carcinoma
IDO: indoleamine 2:3-dioxygenase
ALT: alternative lengthening of telomeres
ECTR: extrachromosomal telomere repeat
ADU: dithio-modified diadenosine
DMXAA: 5:6-dimethylxanthenone-4-acetic acid
T-ALL: T-cell acute lymphoblastic leukaemia
AML: acute myeloid leukemia
CLL: chronic lymphocytic leukemia
ML-RR-CDA: modified synthetic CDN: dithio-(R_P_: R_P_)-[cyclic[A(2’:5′) pA(3′:5′)p]]
5FU: 5-florouracil
PARP: Poly (ADP) ribose polymerase
NHEJ: non-homologous end joining
MMEJ: micro-homology end joining
HR: homologous recombination
PARPi: Poly (ADP) ribose polymerase inhibitor
ICB: immune checkpoint blockade
SIRPα: signal regulatory protein alpha
ATM: ataxia telangiectasia mutated
IFI16: interferon-γ-inducible factor 16
RA: rheumatoid arthritis
SLE: systemic lupus erythematosus
RVCL: retinal vasculopathy with cerebral leukoencephalopathy
AGS: Aicardi-Goutières syndrome
MI: myocardial infarction
